# Supplementary material for: Cytonemes coordinate asymmetric signaling and organization in the Drosophila muscle progenitor niche
Source: Nat Commun. 2022 Mar 4;13:1185. doi: 10.1038/s41467-022-28587-z (PMC8897416; doi:10.1038/s41467-022-28587-z)
Supplement: Supplementary file 1 — Supplementary Information [file 41467_2022_28587_MOESM1_ESM.pdf]

SUPPLEMENTARY INFORMATION

Cytonemes coordinate asymmetric signaling and organization in the *Drosophila* muscle progenitor niche

Akshay Patel[1], Yicong Wu[2], Xiaofei Han[2], Yijun Su[2,3], Tim Maugel[4], Hari Shroff[2,3], Sougata Roy[1*]

[1] Department of Cell Biology and Molecular Genetics, University of Maryland, College Park, Maryland, USA

[2] Laboratory of High-Resolution Optical Imaging, National Institute of Biomedical Imaging and Bioengineering, National Institutes of Health, Bethesda, Maryland, USA

[3] Advanced Imaging and Microscopy Resource, National Institutes of Health, Bethesda, Maryland, USA

[4] Department of Biology, Laboratory for Biological Ultrastructure, University of Maryland, College Park, Maryland, USA

* Correspondence: sougata@umd.edu

This file includes

Supplementary Figures 1-7

Supplementary Tables 1-3

References

Supplementary Figure 1. Characterization of AMP localizations in the wing disc niche.

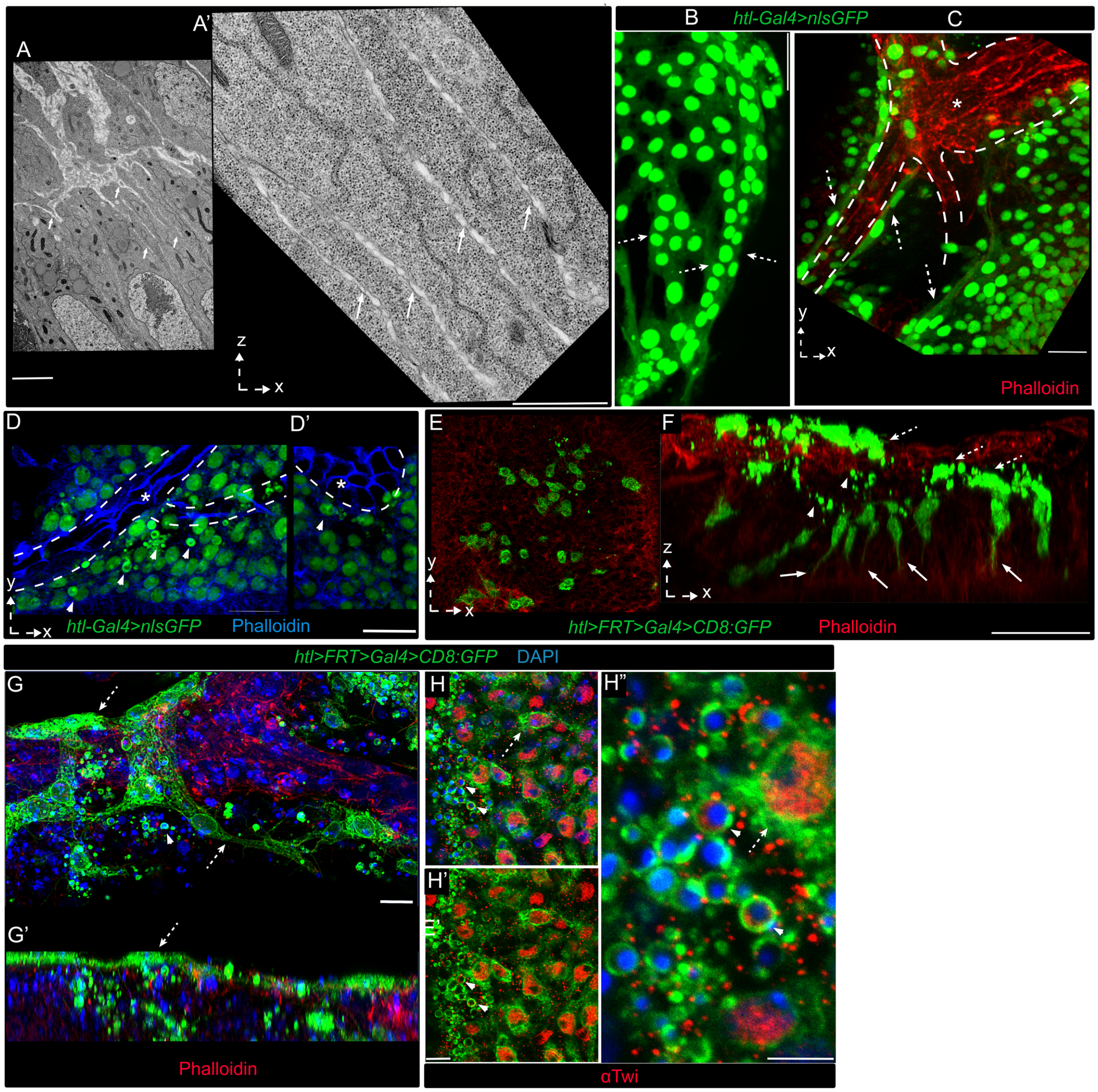

A-A' TEM sections of a w^{1118} wing disc showing cytoneme-like AMP projections within the intercellular space of the wing disc epithelium. **B-D'** XY sections showing diverse morphology of distal layer AMPs; arrowhead, small non-polar cells; dashed arrow, multinucleated elongated cells in juxtaposition to the trachea (*, dashed lines, phalloidin stained) (also see Fig.1I-K). **E-H"** Images of a wing disc harboring CD8:GFP-marked AMP clones; E, F, a single optical XY section; F, YZ view of a wing disc showing orthogonal (arrows) and lateral (dashed arrows) orientation of cells and cytonemes, occurring exclusively in proximal (arrow) and distal (dashed arrow) clones, respectively, relative to the disc plane; G-G', Clones of distal cells showing diverse morphologies, cell-cell adhesion, multi-nucleated assembly; red, phalloidin; H-H", Twi-immunostained tissues showing small spherical cells, large elongated cells; dashed arrow, elongated cells; arrowhead, spherical cells. Scale bars: 20μm; 5μm (A); 2μm (A'); 20μm (B-F); 5μm (G-H").

Supplementary Figure 2. Characterization of AMP cytonemes.

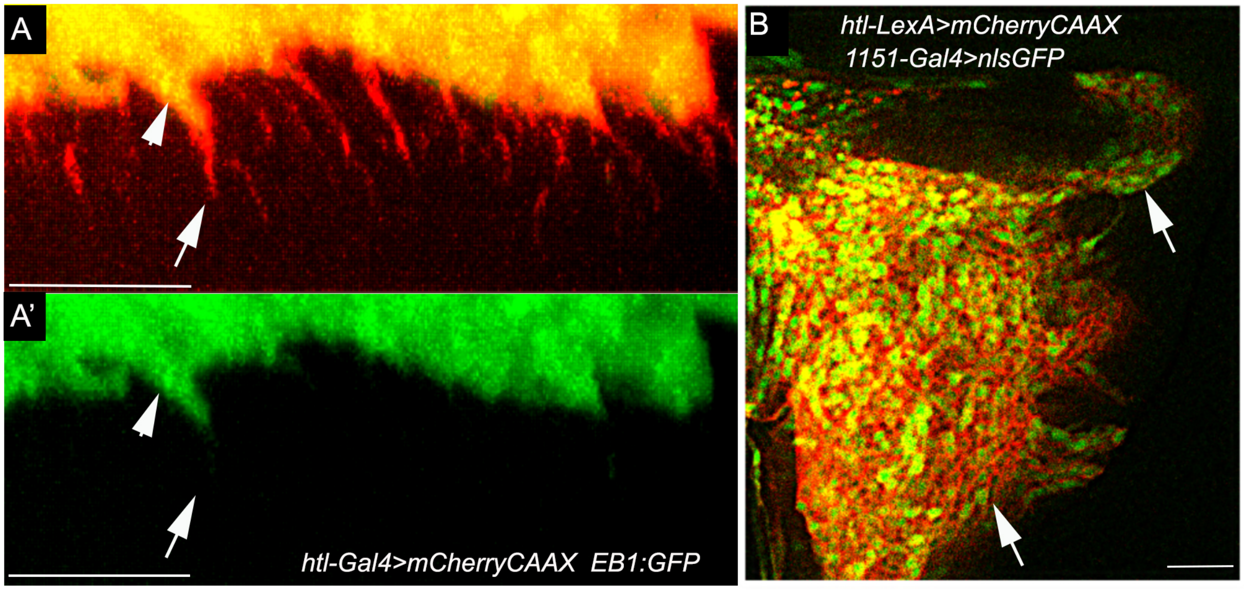

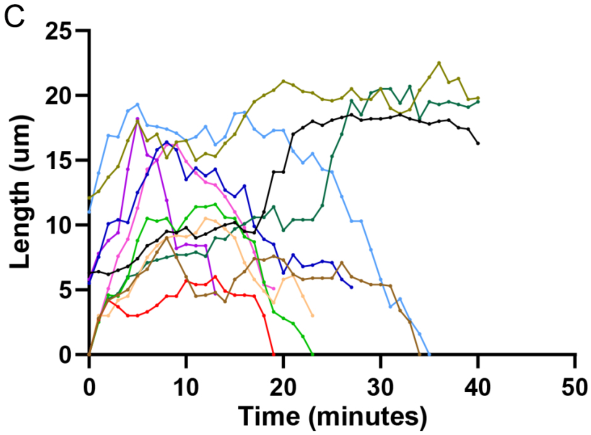

D

htl-LexA>CD2:GFP		# Cytonemes	# of discs
Average Maximum extension (μm)	14.9 ± 4.4	19	4
Average Projection velocity (μm /min)	1.2 ± 0.8	19	4
Average Retraction velocity (μm /min)	0.8 ±0.4	19	4
Average Lifetime (min)	25.07 ±11.3	105	4

A-A' mCherryCAAX-marked AMPs expressing EB1:GFP lack GFP signal at the growing tips of orthogonal cytonemes; Arrowheads, cytonemes base; arrows, cytonemes shaft, and tip. **B** Highly specific pan-AMP expression pattern of *htl-LexA* binary transcription driver as verified by the overlap of expression pattern (arrow) over the known *1151-Gal4* pan-AMP driver. **C** Kymograph showing the dynamics of *htl-LexA>LexO*-CD2:GFP marked AMP cytonemes; each line graph indicates tracking of a single filopodium (4 discs). **D.** Analyses of cytoneme dynamics; average values ± SD were derived from >19 cytonemes in four *ex vivo* cultured wing discs as indicated. Source data are provided as a Source Data file. Scale bars: 20μm.

Supplementary Figure 3. Generation and expression of *pyr-Gal4*.

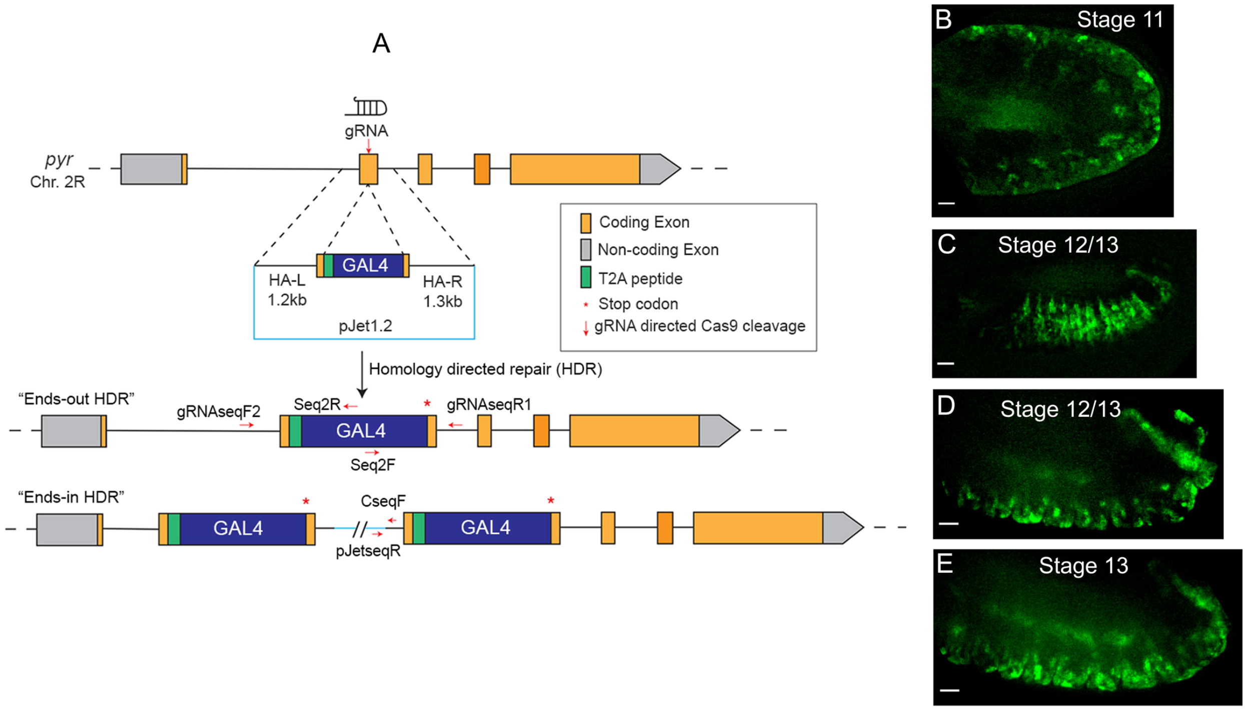

A Schematic illustration of CRISPR/Cas9-based genome editing to generate *pyr-Gal4* transgenic *Drosophila*; indicated primers were used to screen "ends-out" HDR lines (See Materials and Methods). **B-E** Different stages of embryos expressing CD8:GFP under *pyr-Gal4*; *pyr-Gal4* expression patterns matched previously published *pyr* mRNA *in situ* hybridization patterns, e.g., embryonic ectoderm near pericardial cell precursors (B), lateral view of segmental epithelial stripes (C), and ventral epithelial expression near proctodeum and stomodeum (D,E). Scale bars: 50μm.

Supplementary Figure 4. Niche-specific signaling of Pyr:GFP and Ths:GFP.

A

Genotype	# of discs	AMP location	# of AMPs	Av. thickness of ad-epithelial layer (μm)	# of layers
htl>nls:mCherry	3	Hinge	18.7±4.0	5.7±0.4	1.7 ±0.6
		Notum	31.3±3.8	28.7± 4.6	4.7 ±0.6
pyr>Pyr:GFP, htl>nls:mCherry	3	Hinge	35.0±3.6 ***	15.0±1.3 ***	3.7 ±0.6 ***
		Notum area	48.0±11	45±2.2 **	6.7 ±0.6
ths>Ths:GFP, htl>nls:mCherry	3	Hinge	18.0±4.4	6.8±1.4	2.0 ±0.0
		Notum	102.3±19.6 ***	59.4±7.5 ***	13.3±1.5 ***

**, p<0.05; ***, p<0.01 (t-test compared to *htl-LexA*>nls:mCherry).

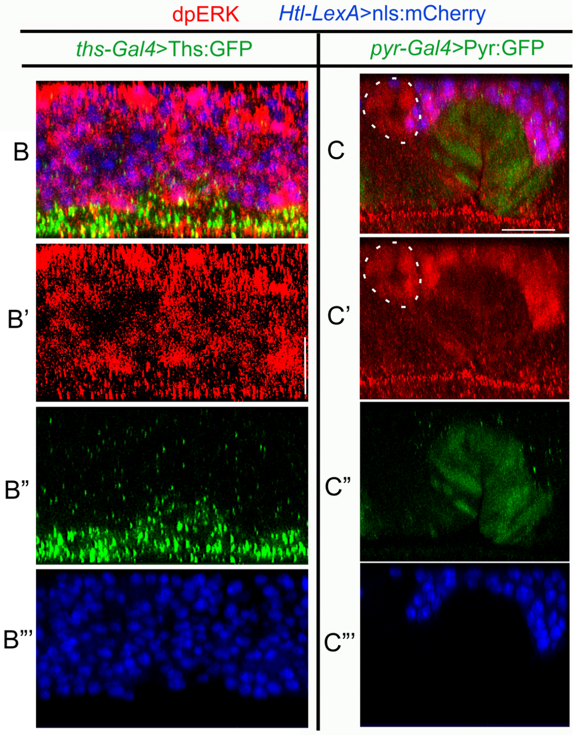

A-C''' Wing discs expressing Pyr:GFP and Ths:GFP under *pyr-Gal4* and *ths-Gal4*, respectively. **A** Niche-specific change in the AMP pool size and stratified organization due to the Pyr:GFP and Ths:GFP expression from their respective sources; hinge, *pyr* source; notum, *ths*-source; Average values ± SD shown; **, p<0.05; ***, p<0.01 (unpaired two-tailed t-test). **B-C'''** Activation of dpERK (red) in all signal-receiving AMPs (blue); dashed circle, ASP. Scale bars: 20μm. Source data are provided as a Source Data file.

Supplementary Figure 5. AMP homing on ectopic Pyr:GFP and Ths:GFP-expressing source.

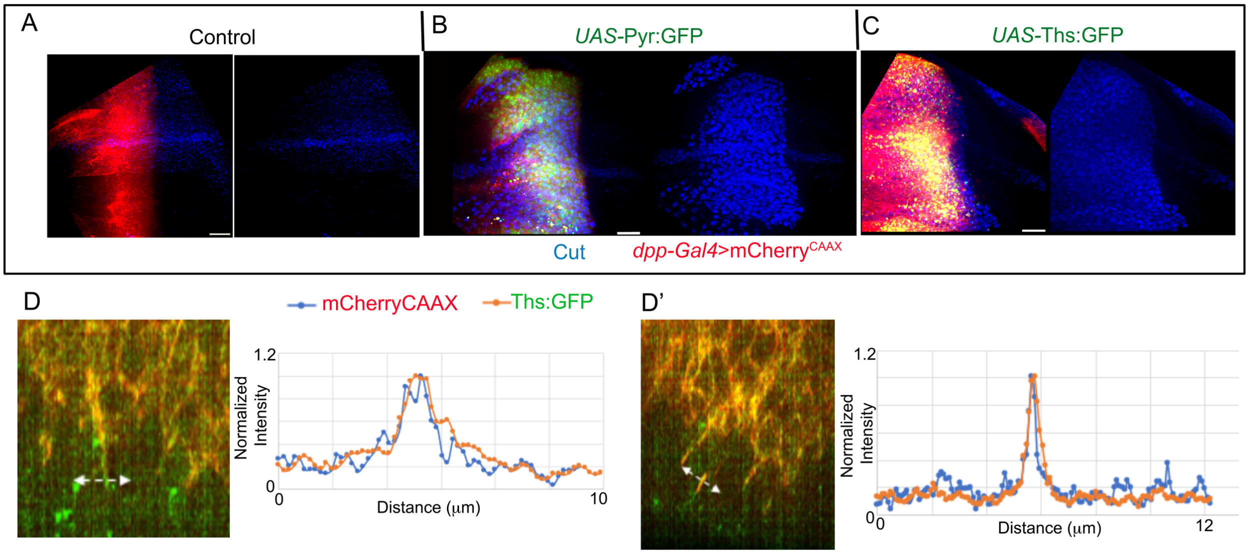

A-C The homing of AMPs (marked with αCut) over the disc *dpp*-source ectopically expressing of Pyr:GFP (B) or Ths:GFP (C); A, control disc region (*dpp-Gal4, UAS-mCherryCAAX/*+). **D-D'** Raw quantitation data from single XZ confocal slices from wing discs to assess colocalization of Ths:GFP produced from the *dpp* source in the disc pouch and mCherryCAAX-marked AMP cytonemes that invade into the ectopic niche. Genotypes: *dpp-Gal4/LexO-mCherryCAAX; htl-LexA/UAS-Ths:GFP* (D, D'). Source data are provided as a Source Data file. Scale bars: 20μm.

Supplementary Figure 6. AMP cytonemes in the Wg-expressing disc source.

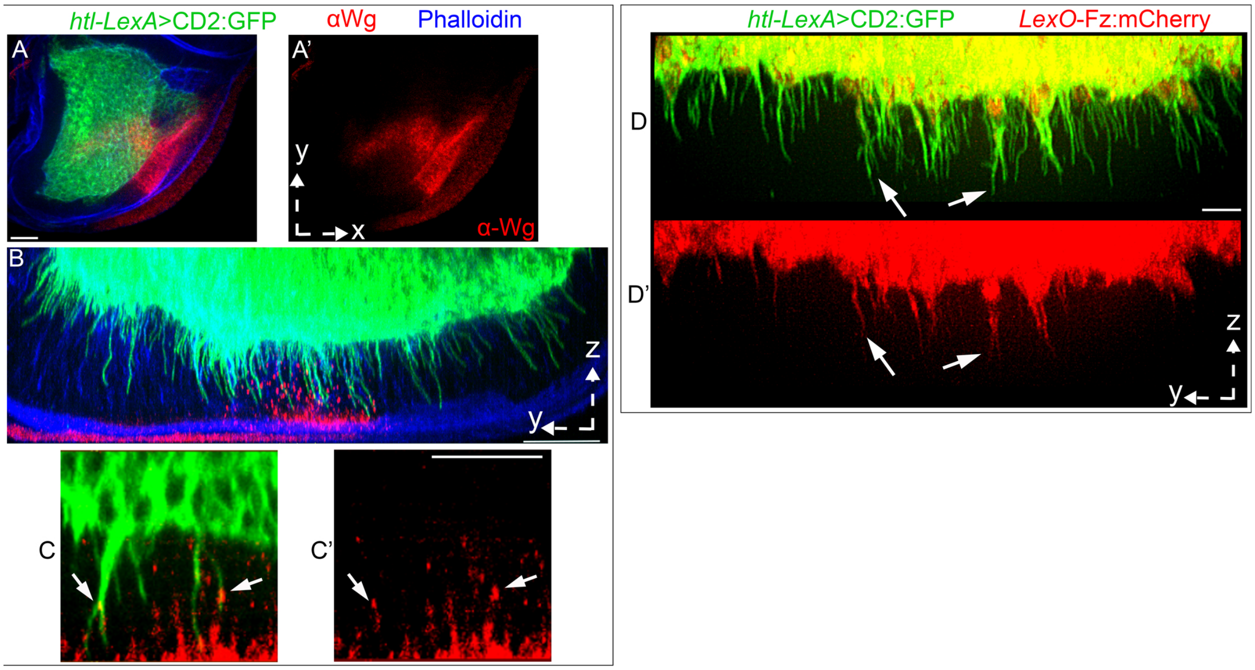

A,A' CD2:GFP-marked AMPs over the Wg-expressing (αWg; red) zone in the wing disc. **B,C** A group of AMP cytonemes that occupied the *ths*-expressing wing disc notum also receive Wg (αWg; red; arrow in C). **D, D'** CD2:GFP-marked AMPs expressing *LexO*-Fz:mCherry under *htl-LexA* showed Fz:Cherry-containing cytonemes specifically within the Wg-expressing zone (arrows). Scale bars: 20μm.

Supplementary Figure 7. Non-autonomous effects of cytoneme-deficient AMPs on the wing disc.

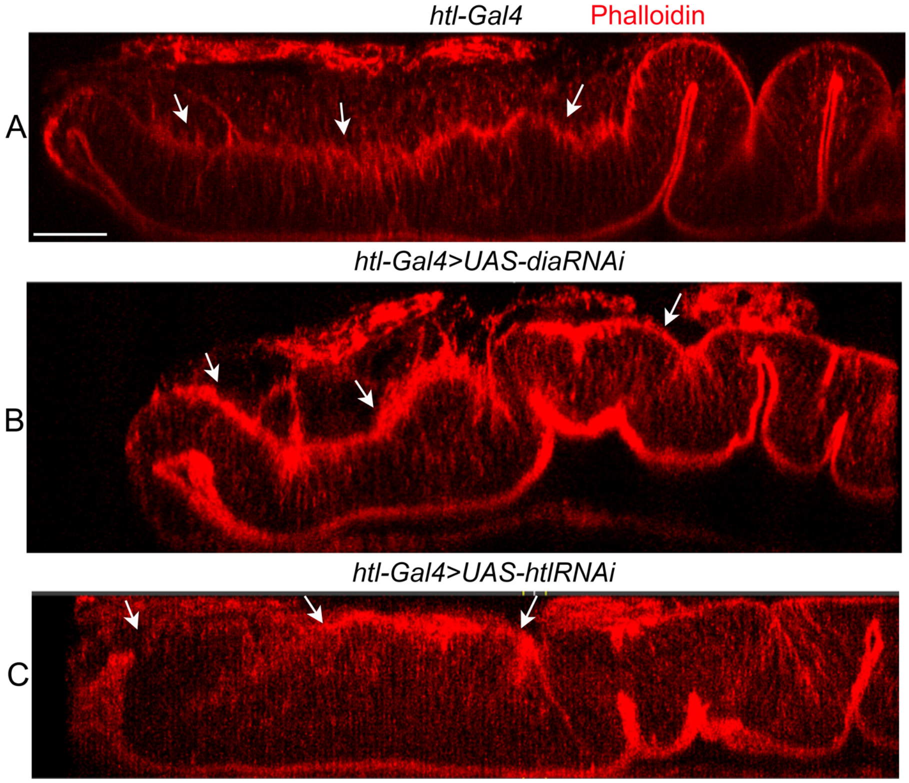

A-C YZ sections of wing imaginal discs, comparing identical region of discs derived from WT control (*htl-Gal4 X* w^{-}) and mutant flies that expressed either *dia-i* (B) or *htl-i* (C) under *htl-Gal4* (*htl-Gal4 X UAS-RNA-i*); red, phalloidin staining; arrows, basal surface of wing disc. Note that *dia-i* expressing syncytial AMPs caused abnormal disc morphology, probably to accommodate large-sized AMPs (also see Figure 4H-I). Similarly, the loss of disc-specific polarity of *htl-i-* expressing AMPs/AMP cytonemes caused smoothened disc surface and reductions in wing disc folds and actin-rich projections (also see Figure 5G-J). Scale bars: 20μm.

SUPPLEMENTARY TABLES

Supplementary Table 1. Polarized AMP morphology and organization in niche

	Genotype	# of discs	AMP layer	Av. major axis length orthogonal or oblique to the disc (μm) ± SD	Av. major axis length parallel to the disc (μm) ±SD	# of AMPs	Angle between major axes of AMPs and disc (av. deg.) ± SD	Av. thickness of the ad-epithelial layer (μm) ± SD	# of layers ± SD
EM	*w* ⁻	2 *	p	7.1±1.5	3.1±0.6	16	81.9±7.8	21.5± 1.5	4.0 ±0.0
			d	2.3±1.1	8.7±2.6	11	11.7±4.8		
Light microscopy	*htl-Gal4> nls:GFP*	5	p	5.5±1.2	2.9±0.6	125	83.4±5.5	26.0±1.2	4.5±0.7
			p^{-1}	4.7±1.1	3.7±0.9	119	77.2±9.5		
			d^{-1}	3.9±1.1	4.3± 0.9	84	21.0±13.1		
			d	3.2±1.1	5.2±1.1	58	8.8±6.0		
	htl-Gal4> nls:GFP, htl-i	3	p	3.1±0.8 **	6.2±1.9 **	33	6.0±4.3 **	5.9±1.3 **	1.0±0.0 ***

htl-Gal4> nls:GFP, dia-i	# of discs	Av. diameter of myoblasts (μm)	Average thickness of myoblasts (μm)	Average nuclei per chamber	Average number of chambers
	5	17.5 ±10.9	50.5 ±12.6	6.5 ±1.2	6.4 ±1.1

Note: p, proximal. d, distal. p^{-1}, one layer above p relative to the disc. d^{-1}, one layer below d relative to the disc. Source data are provided as a Source Data file.

*, 16 TEM sections from 2 wing discs.

**, p <0.0001, unpaired two-tailed t-test compared to *htl>nls:GFP*.

***, p = 0.0002, unpaired two-tailed t-test compared to *htl>nls:GFP*.

Supplementary Table 2. Clonal analyses of AMP and AMP cytonemes

Genotype		# of discs	Clone layer	# Clones *	Av. # disc-directed cytoneme/cell ± SD	Av. # lateral cytoneme/cell ± SD	Av. # of non-polar small cells ± SD	Av. cell angle (av. Deg. ± SD)
htl>FRT>Gal4	*UAS-CD8:GFP*	3	p	41	3.2±0.9	0.0	0	83.2±4.7
			d	15	0	5.7± 1.3	39.7± 21.9	2.5±3.2
	Lifeact:GFP	3	p	25 **	2.6±0.9 **	0	0	85.7±5.3
			d	19	0	5.6± 1.8	36.7± 9.5	1.1±1.2
	Lifeact:GFP, dia-i	9	p	0 **	0 **	ND	0	NA
			d	NA	NA	ND	71.6± 66.3	NA
	Lifeact:GFP, htl-i	10	p	0 **	0 **	ND	0	NA
			d	24	0	6.1± 1.7	57.3± 37.4	0.79±1.2

Note: *, excludes non-polar spherical cells. p, proximal. d, distal. **, $p < 0.01$ (One-way ANOVA followed by Tukey HSD) comparing same myoblast layers between control (*htl>FRT>Gal4>Lifeact:GFP*) and *htl-i and dia-i*. Source data are provided as a Source Data file.

Supplementary Table 3. Reagent list used in this study

REAGENT or RESOURCE	SOURCE	IDENTIFIER
Antibodies		
Mouse anti-MAP Kinase, Activated (Diphosphorylated ERK-1&2) (1:250)	Sigma-Aldrich	Cat# M-8159; RRID:AB_477245
Rabbit anti-PH3 (1:2000)	Cell Signaling Technology	Cat# 9701; RRID:AB_331535
Mouse anti-Cut (1:50)	DSHB	Cat# 2B10; RRID:AB_528186
Mouse monoclonal anti-Discs large (1:100)	DSHB	Cat# 4F3 anti-discs large; RRID: AB_528203
Mouse anti-Armadillo (1:100)	DSHB	Cat# N2 7A1; RRID:AB_528089
Mouse anti-Wingless (1:50)	DSHB	Cat# 4D4; RRID:AB_528512
Rat anti-Shotgun (1:50)	DSHB	Cat# DCAD2; RRID:AB_528120
Rabbit anti-Twist (1:2000)	[1]	N/A
Rabbit anti-Vestigial (1:200)	[2]	N/A
Goat anti-Mouse IgG (H+L), Alexa Fluor 555	Thermo Fisher Scientific	A21434
Goat anti-Mouse IgG (H+L), Alexa Fluor 647	Thermo Fisher Scientific	A28181
Goat anti-Rat IgG (H+L), Alexa Fluor 647	Thermo Fisher Scientific	A21247
Goat anti-Rabbit IgG (H+L), Alexa Fluor 555	Thermo Fisher Scientific	A21428
Goat anti-Rabbit IgG (H+L), Alexa Fluor 647	Thermo Fisher Scientific	A21244
Bacterial and Virus Strains		
DH5 Alpha		
Chemicals, Peptides, and Recombinant Proteins		
Phalloidin iFlor 555	Abcam	Cat# ab176759
Phalloidin iFlor 647	Abcam	Cat# ab176756
Sodium Cacodylate	Electron Microscopy Sciences	Cat# 12300
Osmium Tetroxide	Electron Microscopy Sciences	Cat# 19140
Potassium Ferricyanide	Sigma-Aldrich	Cat# 702587
Uranyl Acetate	Electron Microscopy Sciences	Cat# 22400
Propylene Oxide	Electron Microscopy Sciences	Cat# 20401
Low Viscosity Resin	Electron Microscopy Sciences	Cat# 14300

Lead Citrate	Electron Microscopy Sciences	Cat# 17800
Poly-L-lysine	VWR	Cat# 48393241
Critical Commercial Assays		
CloneJET PCR Cloning Kit	Thermo Fisher Scientific	Cat# K1231
Gateway™ LR Clonase™ II Enzyme mix	Thermo Fisher Scientific	Cat# 11791020
Zymoclean Gel DNA Recovery Kit	Zymo Research	Cat# D4007
GeneJET Plasmid Miniprep Kit	ThermoFisher Scientific	Cat# K0502
GeneJET Plasmid Midiprep Kit	ThermoFisher Scientific	Cat #K0481
2X PCR Premix	Syd Labs	Cat# MB067-EQ2R-L
Deposited Data		
Raw data from all the figures	This paper	
Experimental Models: Organisms/Strains		
D. melanogaster: *UAS-CD8:GFP*	BDSC	5130
D. melanogaster: *UAS-CD8:GFP*	BDSC	5137
D. melanogaster: *UAS-CD8:RFP*	BDSC	32218
D. melanogaster: *UAS-mCherryCAAX*	BDSC	59021
D. melanogaster: *lexO-mCherryCAAX*	[3]	N/A
D. melanogaster: *lexO-CD2:GFP*	BDSC	66544
D. melanogaster: *UAS-Lifeact:GFP*	BDSC	57326
D. melanogaster: *UAS-Eb1:GFP*	BDSC	35512
D. melanogaster: *UAS-nls:GFP*	BDSC	4776
D. melanogaster: *UAS-nls:mCherry*	BDSC	38425
D. melanogaster: *UAS-Dia-GFP*	BDSC	56751
D. melanogaster: *UAS-ΔDAD-Dia-GFP*	BDSC	56752
D. melanogaster: *LexO-nsyb:*GFP_{1-10}*, UAS-CD4:*GFP_{11}	[4]	N/A
D. melanogaster: *UAS-htl-DN*	BDSC	5366
D. melanogaster: *UAS-htlACT*	BDSC	5467
D. melanogaster: *UAS-pyrRNAi*	BDSC	63547
D. melanogaster: *UAS-diaRNAi*	BDSC	33424
D. melanogaster: *htl-Gal4*	BDSC	40669
D. melanogaster: ths-Gal4	BDSC	77475
D. melanogaster: *{nos-Cas9}ZH-2A*	BDSC	54591
D. melanogaster: *hs-Flp*	BDSC	6
D. melanogaster: w^{1118}	BDSC	3605
D. melanogaster: *htl:*GFP^{fTRG}	VDRC	318120
D. melanogaster: *UAS-htlRNAi*	VDRC	6692
D. melanogaster: *UAS-thsRNAi*	VDRC	24536
D. melanogaster: dpp-Gal4	[5]	N/A
D. melanogaster: *LexO-Fz:mCherry*	[5]	N/A
D. melanogaster: *1151-Gal4*	[5]	N/A
D. melanogaster: *htl-LexA*	This paper	N/A
D. melanogaster: *pyr-Gal4*	This paper	N/A
D. melanogaster: *htl>FRT>stop>FRT>Gal4*	This paper	N/A

D. melanogaster: *LexO-Htl:mCherry*	This paper	N/A
D. melanogaster: UAS-Ths:GFP	This paper	N/A
D. melanogaster: *UAS-Pyr:GFP*	This paper	N/A
Oligonucleotides		
Primer for cloning *pyr-Gal4*: AGGACTTATATTATACTGATGGTGAGTTTTTGTCC	This paper	N/A
Primer for cloning *pyr-Gal4*: AATTCGAGCTCGGTACCCTTCTGCTATTGATCTGC CAGCG	This paper	N/A
Primer for cloning *pyr-Gal4*: GCCCAATGTCGAATTCGGCTCCGGCGAAGG ACGCGGCAGCCTACTGACTTGCGGAGATGTCGAA GAG AACCCTGGCCCTATGAAGCTACTGTCTTCTATC	This paper	N/A
Primer for cloning *pyr-Gal4*: CGCCGGAGCCGAATTCGACATTGGGCATGAACTT GTGGAAC	This paper	N/A
Primer for cloning *pyr-Gal4*: GCCAAGCTTGCATGCCTCTAGA TGACATTCTGCAGATACGGGTAGTTC	This paper	N/A
Primer used for *pyr-Gal4* screen: GATCTCACGATCGGCCGTAAAATG	This paper	gRNAseqF2
Primer used for *pyr-Gal4* screen: GATTTCGATTACACACACTCAATCTCTCG	This paper	gRNASeqR1
Primer used for *pyr-Gal4* screen: GGATGCTATTAACCCTGAACTTTC	This paper	pJet seqR
Primer used for *pyr-Gal4* screen: CGCAGGGGATTTCTCC	This paper	CseqF
Primer used for *pyr-Gal4* screen: CCAGATTGAAATCGCG	This paper	Seq2F
Primer used for *pyr-Gal4* screen: CCAATGGCTAATATGCAG	This paper	Seq2R
gRNA for *pyr-Gal4*: ATAATATAAGTCCTGACATTGGG	This paper	N/A
Primer for cloning *htl-enh-FRT-stop-FRT3-FRT-FRT3-Gal4*: AATTCGAGCTCGGTACCGCTAGCGG CAAGGAGAAATTCCAACGCAGAGAC	This paper	N/A
Primer for cloning *htl-enh-FRT-stop-FRT3-FRT-FRT3-Gal4*: GATGAACGGGTGGGGATGG	This paper	N/A
Primer for cloning *htl-enh-FRT-stop-FRT3-FRT-FRT3-Gal4*: CGAGACCGGCACGAGTCTG	This paper	N/A
Primer for cloning *htl-enh-FRT-stop-FRT3-FRT-FRT3-Gal4*: GGGAGATTTAAGAGAGGTAGAGAATC	This paper	N/A
Primer for cloning *htl-enh-FRT-stop-FRT3-FRT-FRT3-Gal4*: TGTTCCAAATTGGTCCGCGTAGTCC	This paper	N/A
Primer for cloning *htl-enh-FRT-stop-FRT3-FRT-FRT3-Gal4*: CTACGCGGACCAATTTGGAACAACCAAACCG AAAGACTTAATTTATATTTATTTAATTAATTTTAATAA AAC	This paper	N/A
Primer for cloning *htl-enh-FRT-stop-FRT3-FRT-FRT3-Gal4*: GGCAAAAAAAAAACTGAGAATTTGC	This paper	N/A
Primer for cloning *htl-enh-FRT-stop-FRT3-FRT-FRT3-Gal4*: GCCAAGCTTGCATGCCCTCCTCTATGCCTGAACCC AGC	This paper	N/A

Primer for cloning *htl-LexA*: CACCGCAAGGAGAAATTCCAACGCAGAGAC	This paper	N/A
Primer for cloning *htl-LexA*: GCCAAGCTTGGCGAATTCTGTTCCAAATTGGTCCG CGTAGTCC	This paper	N/A
Primer for cloning *UAS-Htl:mcherry:* AATTCGAGCTCGGTACCGAATTCATGGCTGCCGCC TGG	This paper	N/A
Primer for cloning *UAS-Htl:mcherry:* GGATCTGATCAAATTTGCCCACC	This paper	N/A
Primer for cloning *UAS-Htl:mcherry:* CTGACGAGCACATTCCTGGC	This paper	N/A
Primer for cloning *UAS-Htl:mcherry:* GCCAAGCTTGCATGCCCTCGAGAT AATTACACCACTTCTGCAGGTTGTCC	This paper	N/A
Primer for cloning *UAS-Pyr:GFP*: AATTCGAGCTCGGTACCGCGGCCG CATGTTCCACAAGTTCATGCCCAATG	This paper	N/A
Primer for cloning *UAS-Pyr:GFP*: AGCTCCTCGCCCTTGGACATG GTTGTTGTGGTTGTTGTTGTTGTG	This paper	N/A
Primer for cloning *UAS-Pyr:GFP*: CAACAACAACCACAACAACCATGTCCAAGGGCGA GGAGC	This paper	N/A
Primer for cloning *UAS-Pyr:GFP*: GCCAAGCTTGCATGCGCTAGC TGGTGTCTTGTACAGCTCATCCATGCCC	This paper	N/A
Primer for cloning *UAS-Pyr:GFP*: GGCATGGATGAGCTGTACAAGACACCAGCTAGCC CAGTGG	This paper	N/A
Primer for cloning *UAS-Pyr:GFP*: GCCAAGCTTGCATGCCGGATCCCTCGAGCTATAA ATCTATATAATACAAGCTAACAAAATACTTACCAC	This paper	N/A
Primer for cloning *UAS-Ths:GFP*: AATTCGAGCTCGGTACCGCGGCCGCAT GTCGAATCAGTTAGAGAGACTGCTG	This paper	N/A
Primer for cloning *UAS-Ths:GFP*: GCCGCCTTGCCCCTCGACGCTCTTCTTGGGCCCC ACAG	This paper	N/A
Primer for cloning *UAS-Ths:GFP*: GTCGAGGGGCAAGGCGGCATGTCCAAGGGCGAG GAGC	This paper	N/A
Primer for cloning *UAS-Ths:GFP*: ATGTCCAAGGGCGAGGAGC	This paper	N/A
Primer for cloning *UAS-Ths:GFP*: ACTGCCGCCGCCACTGCCCTTGTACAGCTCATCCA TGCCC	This paper	N/A
Primer for cloning *UAS-Ths:GFP*: GGCAGTGGCGGCGGCAGTGTCAACGATGCCTGCT ACATGTTC	This paper	N/A
Primer for cloning *UAS-Ths:GFP*: GCCAAGCTTGCATGCCTCTAGAC TACGCAAATCTCTGATGAGTGAACC	This paper	N/A
Recombinant DNA		
pUC19	Addgene	50005

pUASt	DGRC	1000
pACT-FRT-stop-FRT3-FRT-FRT3-Gal4	Addgene	52889
pBPnlsLexA::p65Uw	Addgene	26230
pCFD3	[6]	N/A
pCFD3-pyr-Gal4-gRNA	This paper	N/A
pJet1.2-pyr-T2A-Gal4	This paper	N/A
pLot-Htl:mCherry	This paper	N/A
pHtl-enh-FRT-stop-FRT3-FRT-FRT3-Gal4	This paper	N/A
pBP-htl-enh-nlsLexA::p65Uw	This paper	N/A
p{GMR93H07-Gal4}	[7]	N/A
Software and Algorithms		
Fiji- ImageJ 1.52p	ImageJ	https://fiji.sc
Prism 8.0	GraphPad	https://www.graphpad.com/
Adobe Photoshop 22.5.1	Adobe	https://www.adobe.com
Adobe Illustrator 25.4.1	Adobe	https://www.adobe.com
Microsoft Excel (Version 2111)	Microsoft	https://www.office.com
SnapGene 3.3.4	SnapGene	https://www.snapgene.com
VassarStats		vassarstats.net
Imaris 9.5.0	Imaris	https://imaris.oxinst.com
Matlab 2019b	MathWorks	https://mathworks.com
Andor iQ3	Oxford Instruments	https://andor.oxinst.com/products/iq-live-cell-imaging-software/
Zen 3	Carl Zeiss Microscopy GmbH	https://www.zeiss.com/microscopy/int/home.html?vaURL=www.zeiss.com/microscopy

REFERENCES

1. Roth, S., Stein, D. & Nüsslein-Volhard, C. A gradient of nuclear localization of the dorsal protein determines dorsoventral pattern in the Drosophila embryo. *Cell* **59,** 1189–1202 (1989).
2. Williams, J. A., Bell, J. B. & Carroll, S. B. Control of Drosophila wing and haltere development by the nuclear vestigial gene product. *Genes & Development* **5,** 2481–2495 (1991).

3. Roy, S., Huang, H., Liu, S. & Kornberg, T. B. Cytoneme-mediated contact-dependent transport of the Drosophila decapentaplegic signaling protein. *Science* **343,** 1244624–1244624 (2014).
4. Du, L., Sohr, A., Yan, G. & Roy, S. Feedback regulation of cytoneme-mediated transport shapes a tissue-specific FGF morphogen gradient. (2018). doi:10.7554/eLife.38137
5. Huang, H. & Kornberg, T. B. Myoblast cytonemes mediate Wg signaling from the wing imaginal disc and Delta-Notch signaling to the air sac primordium. *Elife* **4,** e06114 (2015).
6. Port, F., Chen, H. M., Lee, T. & Bullock, S. L. *An optimized CRISPR/Cas toolbox for efficient germline and somatic genome engineering in Drosophila*. (2014). doi:10.1101/003541
7. Pfeiffer, B. D. *et al.* Tools for neuroanatomy and neurogenetics in Drosophila. *Proc. Natl. Acad. Sci. U.S.A.* **105,** 9715–9720 (2008).
