# Supplementary material for: Cytonemes coordinate asymmetric signaling and organization in the Drosophila muscle progenitor niche
Source: Nat Commun. 2022 Mar 4;13:1185. doi: 10.1038/s41467-022-28587-z (PMC8897416; doi:10.1038/s41467-022-28587-z)
Supplement: Supplementary file 3 — Description of Additional Supplementary Files [file 41467_2022_28587_MOESM3_ESM.pdf]

Description of Additional Supplementary Files

File Name: Supplementary Movie 1
Description: **3D-rendered view of orthogonal AMP cytonemes visualized using live imaging**. The image was captured from the tip of the cytonemes to the ventral sides of the AMPs. Genotype: *htl-LexA, LexOCD2:GFP*. Scale bar: 10μm (between two long ruler marks)

File Name: Supplementary Movie 2
Description: **A time-lapse movie showing dynamics of AMP cytonemes in ex vivo cultured wing discs.** The time-lapse interval is 1 min. Genotype: *htl-LexA, LexO-CD2:GFP.* Scale bar: 5μm (between two long ruler marks).

File Name: Supplementary Movie 3
Description: **Time-lapse movie showing growth of AMP cytonemes.** The time-lapse interval is 1 min. Genotype: *htl-LexA, LexOCD2:GFP*. Scale bar: 5μm (between two long ruler marks).

File Name: Supplementary Movie 4
Description: **Dynamic niche-sharing by multiple AMP cytonemes over time**. Time-lapse (1 min intervals) movie of a developing wing disc in ex vivo culture showing dynamic niche sharing by AMP cytonemes. Genotype: *htl-LexA, LexOCD2:GFP*.

File Name: Supplementary Movie 5
Description: **3D-reconstruction of a wing disc showing the organization of orthogonal AMP cytonemes within the disc epithelium.** The 3D image was captured using a triple-view confocal microscope and processed as described in Methods. Genotype: *ths-Gal4/ UASnls:mCherry; htl-LexA/ LexO-CD2:GFP*. Color channels: AMP cytonemes (red) disc cell nuclei (blue) are pseudo-colored.

File Name: Supplementary Movie 6
Description: **Serial YZ stacks, showing AMP cytonemes (green) invading through the basolateral inter-cellular space between *ths*-expressing disc cells (red).** The nanoscopic resolution was achieved using Airyscan microscopy. Genotype: *ths-Gal4, UASmCherryCAAX/+ ; htl-LexA, LexOCD2:GFP/+*. Scale bar: 2μm.

File Name: Supplementary Movie 7
Description: **Synaptic contacts between AMP cytonemes and adherens junctions of the wing disc epithelium.** A 3D rendered view showing AMP cytonemes (green) grew between the intercellular space of wing disc cells (unmarked) and contacted Dlg-marked apical adherens junction of the wing disc epithelium (blue). The gap between the two Dlg-stained membranes represents the luminal space of the sac-like wing disc epithelium. Genotype: *thsGal4, UAS-mCherryCAAX/+; htl-LexA, LexO-CD2:GFP/+*. Scale bar: 1μm.

File Name: Supplementary Movie 8
Description: **Endogenous Htl:GFP^{fTRG} on niche-occupying AMP cytonemes.** 3D projection of image volume showing disc-invading AMP cytonemes localizing endogenous Htl:GFP^{fTRG} puncta. Wing disc unmarked and on the left of marked AMPs. Both wing disc hinge and notum areas are shown. Genotype: *htl:GFP*^{fTRG}. Scale bar: 20μm.
