# Supplementary material for: Cytonemes coordinate asymmetric signaling and organization in the Drosophila muscle progenitor niche
Source: Nat Commun. 2022 Mar 4;13:1185. doi: 10.1038/s41467-022-28587-z (PMC8897416; doi:10.1038/s41467-022-28587-z)
Supplement: Supplementary file 12 — Reporting Summary [file 41467_2022_28587_MOESM12_ESM.pdf]

nature portfolio

Corresponding author(s): Sougata Roy

Last updated by author(s): Jan 23, 2022

Reporting Summary

Nature Portfolio wishes to improve the reproducibility of the work that we publish. This form provides structure for consistency and transparency in reporting. For further information on Nature Portfolio policies, see our Editorial Policies and the Editorial Policy Checklist.

Statistics

For all statistical analyses, confirm that the following items are present in the figure legend, table legend, main text, or Methods section.

n/a	Confirmed	
☐	☒	The exact sample size (*n*) for each experimental group/condition, given as a discrete number and unit of measurement
☐	☒	A statement on whether measurements were taken from distinct samples or whether the same sample was measured repeatedly
☐	☒	The statistical test(s) used AND whether they are one- or two-sided<br>*Only common tests should be described solely by name; describe more complex techniques in the Methods section.*
☒	☐	A description of all covariates tested
☒	☐	A description of any assumptions or corrections, such as tests of normality and adjustment for multiple comparisons
☐	☒	A full description of the statistical parameters including central tendency (e.g. means) or other basic estimates (e.g. regression coefficient) AND variation (e.g. standard deviation) or associated estimates of uncertainty (e.g. confidence intervals)
☐	☒	For null hypothesis testing, the test statistic (e.g. *F*, *t*, *r*) with confidence intervals, effect sizes, degrees of freedom and *P* value noted<br>*Give P values as exact values whenever suitable.*
☒	☐	For Bayesian analysis, information on the choice of priors and Markov chain Monte Carlo settings
☒	☐	For hierarchical and complex designs, identification of the appropriate level for tests and full reporting of outcomes
☒	☐	Estimates of effect sizes (e.g. Cohen's *d*, Pearson's *r*), indicating how they were calculated

Our web collection on statistics for biologists contains articles on many of the points above.

Software and code

Policy information about availability of computer code

Data collection	Image data were visualization and analyzed in Andor iQ3 (for Yokogawa CSUX1 spinning disc confocal), in Zen 3 for Zeiss LSM900/Aireyscan 2; FIJI/ImageJ1.52p (https://fiji.sc) and Imaris Imaris 9.5.0 (Bitplane AG) for 3D views, movies and image processing. Sequence data were visualized and analyzed with SnapGene 3.3.4. Triple-view confocal data was analyzed using Matlab 2019b (https://mathworks.com), FIJI/ImageJ1.52p (https://fiji.sc) and Imaris 9.5.0 (Bitplane AG). Adobe Photoshop 22.5.1 and Adobe Illustrator 25.4.1 were used to assemble figure panels. The code for triple-view image/movie reconstruction available at the following link: https://github.com/hroi-aim/multiviewSR.
Data analysis	Statistical analyses were performed using VassarStat (vassarstats.net) and GraphPad Prism 8, MS Excel version 2111. P values were determined either using the unpaired two-tailed t-test for pair-wise comparisons or one way ANOVA followed by Tukey's honestly significant different (HSD) test for comparison of multiple groups. The sample size (N) for each data analysis is indicated in the figures/figure legends/methods/source. P and r values, range of distribution of numerical data and standard deviations are either shown in the respective Figure panels/Tables or are described in the figure legends, materials and methods, and source files.

For manuscripts utilizing custom algorithms or software that are central to the research but not yet described in published literature, software must be made available to editors and reviewers. We strongly encourage code deposition in a community repository (e.g. GitHub). See the Nature Portfolio guidelines for submitting code & software for further information.

Data

Policy information about availability of data

All manuscripts must include a data availability statement. This statement should provide the following information, where applicable:

- Accession codes, unique identifiers, or web links for publicly available datasets
- A description of any restrictions on data availability
- For clinical datasets or third party data, please ensure that the statement adheres to our policy

All data generated and analyzed are included in the manuscript and supporting files. Source data are provided with this paper.

Field-specific reporting

Please select the one below that is the best fit for your research. If you are not sure, read the appropriate sections before making your selection.

☒ Life sciences ☐ Behavioural & social sciences ☐ Ecological, evolutionary & environmental sciences

For a reference copy of the document with all sections, see nature.com/documents/nr-reporting-summary-flat.pdf

Life sciences study design

All studies must disclose on these points even when the disclosure is negative.

Sample size	No statistical method was used to predetermine sample size. The sample size was based on the current standard in the field as well as previous experimental experience. The information about the number of independent replicates, sample size, methods of group allocation, and statistical analyses are mentioned in the main text, methods, figure legends, supplementary tables and Source Data.
Data exclusions	No data were excluded
Replication	The number of independent replicates are mentioned in the texts, Figure/Table legends, and Materials and Methods sections. At least three independent experimental replicates used.
Randomization	Random samples were selected for each group and the number of random samples used is mentioned in the Figures/Tables/Methods/Texts. Same genetic background was used to compare between the control and the test animals. Mosaic analyses include control and test areas within the same organ of an animal. The number of independent animals used for validating the data were mentioned in the relevant text, Figures/tables. The methods section, figure/table legends, and the texts describe the group allocation.
Blinding	In all genetic experiments, the genotype needed to be determined based on different fly genetic/chromosome markers, so blinding was not employed. Samples were selected randomly and unbiased manner. For comparative analyses, internal control and normalized values were compared in an unbiased manner and differences between the control and experimental conditions were highly notable and reproducible in both biological and technical replicates.

Reporting for specific materials, systems and methods

We require information from authors about some types of materials, experimental systems and methods used in many studies. Here, indicate whether each material, system or method listed is relevant to your study. If you are not sure if a list item applies to your research, read the appropriate section before selecting a response.

Materials & experimental systems

n/a	Involved in the study	
☐	☒	Antibodies
☒	☐	Eukaryotic cell lines
☒	☐	Palaeontology and archaeology
☐	☒	Animals and other organisms
☒	☐	Human research participants
☒	☐	Clinical data
☒	☐	Dual use research of concern

Methods

n/a	Involved in the study	
☒	☐	ChIP-seq
☒	☐	Flow cytometry
☒	☐	MRI-based neuroimaging

Antibodies

Antibodies used	Mouse anti-Dlg (1:100; DSHB, Cat# 4F3; RRID: AB_528203); Mouse anti-MAP kinase, Activated (Diphosphorylated ERK-1&2) antibody (1:250; Sigma Aldrich; Cat# M-8159; RRID: AB_477245); Rabbit anti-PH3 (1:2000; Cell Signaling Technology, Cat# 9701, RRID: AB_331535); Mouse anti-Cut (1:50; DSHB, Cat# 2B10, RRID: AB_528186); Mouse anti-Armadillo (1:100; DSHB, Cat# N2 7A1, RRID: AB_528089); Mouse anti-Wingless (1:50; DSHB, Cat# 4D4, RRID: AB_528512); Rat anti-shotgun (1:50; DSHB, Cat# DCAD2, RRID:

AB_528120); Rabbit anti-Twist (1:2000; Roth et. al., Cell, 1989); Rabbit anti-Vestigial (1:200; Williams et. al., Genes and Development, 1991); Goat anti-Mouse IgG (H+L) Alexa Fluor 555/647 (1:1000; Thermo Fisher Scientific; A21434/A28181); Goat anti-Rat IgG (H+L) Alexa Fluor 647 (1:1000; Thermo Fisher Scientific; A21247); Goat anti-Rabbit IgG (H+L) Alexa Fluor 555/647 (1:1000; Thermo Fisher Scientific; A21428/A21244). All dilutions are provided in Supplementary Table 3.

Validation

Anti-twist antibody was validated and published in Roth et. al., Cell, 1989. Anti-Vestigial antibody was validated and published in Williams et. al., Genes and Development, 1991. The following antibodies are form commercial sources and have been validated by the vendors:
(1) Mouse anti-Dlg (1:100; DSHB, Cat# 4F3; RRID: AB_528203)
(a) Vendor website: https://dshb.biology.uiowa.edu/4F3-anti-discs-large
(b) Vendor Validation statement: https://dshb.biology.uiowa.edu/core/media/media.nl?id=1523553&c=571578&h=O22gYwGyD6-6QmGzY5i_W20gahwIwy1BpZJrZUXkQ2mjAtd1&_xt=.pdf

(2) Mouse anti-MAP kinase, Activated (Diphosphorylated ERK-1&2) antibody (1:250; Sigma Aldrich; Cat# M-8159; RRID: AB_477245)
(a) Vendor website: https://www.sigmaaldrich.com/US/en/product/sigma/m8159
(b) Vendor Validation statement: The antibody reacts specifically with the diphosphorylated form of MAP kinase (ERK-1 and ERK-2). It does not recognize the non-phosphorylated or the monophosphorylated forms of MAP kinase or the diphosphorylated forms of JNK and p38 MAP kinase. The epitope recognized by the antibody contains the phosphorylated threonine and tyrosine residues within the regulatory site of active MAP kinase.

(3) Rabbit anti-PH3 (1:2000; Cell Signaling Technology, Cat# 9701, RRID: AB_331535)
(a) Vendor website: https://www.cellsignal.com/products/primary-antibodies/phospho-histone-h3-ser10-antibody/9701
(b) Vendor Validation statement: Phospho-Histone H3 (Ser10) Antibody detects endogenous levels of histone H3 only when phosphorylated at Ser10; however, this antibody does not detect phosphorylated Ser10 when Lys9 is acetylated or methylated. This antibody does not cross-react with histone H3 phosphorylated at Ser28.

(4) Mouse anti-Cut (1:50; DSHB, Cat# 2B10, RRID: AB_528186)
(a) Vendor website: https://dshb.biology.uiowa.edu/2B10
(b) Vendor Validation statement: https://dshb.biology.uiowa.edu/core/media/media.nl?id=1523553&c=571578&h=O22gYwGyD6-6QmGzY5i_W20gahwIwy1BpZJrZUXkQ2mjAtd1&_xt=.pdf

(5) Mouse anti-Armadillo (1:100; DSHB, Cat# N2 7A1, RRID: AB_528089)
(a) Vendor website: https://dshb.biology.uiowa.edu/N2-7A1-Armadillo
(b) Vendor Validation statement: https://dshb.biology.uiowa.edu/core/media/media.nl?id=1523553&c=571578&h=O22gYwGyD6-6QmGzY5i_W20gahwIwy1BpZJrZUXkQ2mjAtd1&_xt=.pdf

(6) Mouse anti-Wingless (1:50; DSHB, Cat# 4D4, RRID: AB_528512)
(a) Vendor website: https://dshb.biology.uiowa.edu/4D4
(b) Vendor Validation statement: https://dshb.biology.uiowa.edu/core/media/media.nl?id=1523553&c=571578&h=O22gYwGyD6-6QmGzY5i_W20gahwIwy1BpZJrZUXkQ2mjAtd1&_xt=.pdf

(7) Rat anti-shotgun (1:50; DSHB, Cat# DCAD2, RRID: AB_528120)
(a) Vendor website: https://dshb.biology.uiowa.edu/DCAD2
(b) Vendor Validation statement: https://dshb.biology.uiowa.edu/core/media/media.nl?id=1523553&c=571578&h=O22gYwGyD6-6QmGzY5i_W20gahwIwy1BpZJrZUXkQ2mjAtd1&_xt=.pdf

(8) Goat anti-Mouse IgG (H+L) Alexa Fluor 555/647 (1:1000; Thermo Fisher Scientific; A21434/A28181)
(a) Vendor website: https://www.thermofisher.com/antibody/product/Goat-anti-Mouse-IgG-H-L-Secondary-Antibody-Recombinant-Polyclonal/A28181
(b) Vendor Validation statement: Anti-Mouse secondary antibodies are affinity-purified antibodies with well-characterized specificity for mouse immunoglobulins and are useful in the detection, sorting or purification of its specified target. Secondary antibodies offer increased versatility enabling users to use many detection systems (e.g. HRP, AP, fluorescence). They can also provide greater sensitivity through signal amplification as multiple secondary antibodies can bind to a single primary antibody. Most commonly, secondary antibodies are generated by immunizing the host animal with a pooled population of immunoglobulins from the target species and can be further purified and modified (i.e. immunoaffinity chromatography, antibody fragmentation, label conjugation, etc.) to generate highly specific reagents.

(9) Goat anti-Rat IgG (H+L) Alexa Fluor 647 (1:1000; Thermo Fisher Scientific; A21247)
(a) Vendor website: https://www.thermofisher.com/antibody/product/Goat-anti-Rat-IgG-H-L-Cross-Adsorbed-Secondary-Antibody-Polyclonal/A-21247
(b) Vendor Validation statement: Anti-Rat secondary antibodies are affinity-purified antibodies with well-characterized specificity for rat immunoglobulins and are useful in the detection, sorting or purification of its specified target. Secondary antibodies offer increased versatility enabling users to use many detection systems (e.g. HRP, AP, fluorescence). They can also provide greater sensitivity through signal amplification as multiple secondary antibodies can bind to a single primary antibody. Most commonly, secondary antibodies are generated by immunizing the host animal with a pooled population of immunoglobulins from the target species and can be further purified and modified (i.e. immunoaffinity chromatography, antibody fragmentation, label conjugation, etc.) to generate highly specific reagents.

(10) Goat anti-Rabbit IgG (H+L) Alexa Fluor 555/647 (1:1000; Thermo Fisher Scientific; A21428/A21244)
(a) Vendor website: https://www.thermofisher.com/antibody/product/Goat-anti-Rabbit-IgG-H-L-Cross-Adsorbed-Secondary-Antibody-Polyclonal/A-21428
(b) Vendor Validation statement: Anti-Rabbit secondary antibodies are affinity-purified antibodies with well-characterized specificity for rabbit immunoglobulins and are useful in the detection, sorting or purification of its specified target. Secondary antibodies offer increased versatility enabling users to use many detection systems (e.g. HRP, AP, fluorescence). They can also provide greater sensitivity through signal amplification as multiple secondary antibodies can bind to a single primary antibody. Most commonly, secondary antibodies are generated by immunizing the host animal with a pooled population of immunoglobulins from the target

species and can be further purified and modified (i.e. immunoaffinity chromatography, antibody fragmentation, label conjugation, etc.) to generate highly specific reagents.

Animals and other organisms

Policy information about studies involving animals; ARRIVE guidelines recommended for reporting animal research

Laboratory animals	Drosophila melanogaster (w-) male and female larvae and adults were used in this study. Trangenic lines were produced using w-strain. Transgenic strains generated in this study: htl-LexA, htl>FRT>stop>FRT>Gal4, UAS-Pyr:GFP, UAS-Ths:GFP and LexO-Htl:mCherry. Genome edited strain generated in this study: pyr-Gal4/CyO. Other strains and sources: Bloomington Drosophila Stock Center: UAS-CD8:GFP (5130, 5137), UAS-CD8:RFP (32218), UAS-mCherryCAAX (59021), LexO-CD2:GFP (66544), UAS-Eb1:GFP (35512), UAS-Lifeact:GFP (57326), UAS-nls:GFP (4776), UAS-nls:mCherry (38425), htl-Gal4 (40669), ths-Gal4/CyO (77475), UAS-Dia:GFP (56751), UAS-delta-DAD-Dia:GFP (56752), UAS-pyrRNAi (63547), UAS-diaRNAi (33424), hs-Flp (6), {nos-Cas9}ZH-2A (54591) and w1118 (3605). Vienna Drosophila Resource Center: htl:GFPfTRG (318120), UAS-htlRNAi (6692), and UAS-thsRNAi (24536). LexO-nsyb:GFP1-10, UAS-CD4:GFP11 from Du et. al., Elife, 2018; LexO-mCherryCAAX from Roy et. al., Science, 2014; dpp-Gal4/CyO, LexO-Fz:mCherry and 1151-Gal4 from Huang, H., and Kornberg, T. B., Elife, 2015.
Wild animals	The study did not involve wild animals.
Field-collected samples	The study did not involve field-collected samples
Ethics oversight	All animal work was done following the University guidelines and the study did not require any additional ethical approval.

Note that full information on the approval of the study protocol must also be provided in the manuscript.
